# Editorial: Cancer stem cell differentiation: A realistic potential therapeutic option?

**DOI:** 10.3389/fonc.2023.1188765

**Published:** 2023-03-31

**Authors:** Mariachiara Buccarelli, Simone Beninati, Claudio Tabolacci

**Affiliations:** ^1^ Department of Oncology and Molecular Medicine, Istituto Superiore di Sanità, Rome, Italy; ^2^ Department of Biology, University of Rome “Tor Vergata”, Rome, Italy

**Keywords:** cancer stem cells, cell plasticity, differentiation markers, trans-differentiation, therapy resistance

During cancer progression, tumor cells undergo molecular and phenotypic changes, collectively referred to as cellular plasticity. Cell plasticity is one of the fundamental abilities of cells to change their properties reversibly. It plays a critical role in different physiological processes, as well as in the evolution and progression of multiple diseases, particularly cancer. Tumor cell plasticity is regulated by highly integrated and complex interactions between endogenous and exogenous stimuli. Understanding the mechanisms underlying the various forms of cell plasticity may deliver new strategies for targeting the most lethal aspects of cancer. In this context, phenotype switching contributes to high heterogeneity, therapeutic resistance, and clonal long-term maintenance within the tumor (1). Key players in these events are cancer cells with stem-like properties (cancer stem cells, CSCs), endowed with an enormous potential for phenotype plasticity, differentiation, and self-renewal capability. Indeed, CSCs have the capacity to self-renew, to give rise to progeny that are different from them, and to utilize common signaling pathways. It is noteworthy that CSCs can trans-differentiate towards different cell lineages, to acquire a more aggressive and therapy-resistant phenotype (2). In the last years, the idea that several cancers might have a subpopulation of self-renewing cells that sustain the development, metastases, drug resistance, and recurrence of tumors has emerged. Several shreds of evidence demonstrated that targeting cell surface markers and stemness-related pathways of CSCs, represents an emerging therapeutic approach (3). The possibility to impair CSC properties through the induction of differentiation represents an interesting and promising anticancer strategy, to overcome therapy resistance.

Within this Research Topic we have collected seven research articles and one review that address the role of CSCs as potential target of therapeutic option.

One of the deadliest cancer types with a poor prognosis is the hepatocellular carcinoma (HCC). Surgical resection is potentially curative, if diagnosed early, while patients with advanced disease are often inoperable. To date, treatments for HCC include tyrosine kinase inhibitors (TKIs) (such as sorafenib, lenvatinib, cabozantinib, and regorafenib) and immune checkpoint inhibitors (ICIs) (such as nivolumab, pembrolizumab, atezolizumab, durvalumab, and ipilimumab). However, acquired drug resistance and the resulting high rate of recurrence are unavoidably developed, probably due to the presence of CSC population, which can be identified based on specific differentiation markers such as CD44, the epithelial cell adhesion molecule (EpCAM), and CD133 (4). Therefore, the use of therapies for preventing HCC relapse by targeting liver CSCs is of great interest. Tang et al. clearly demonstrated that small ubiquitin-like modifier specific peptidase 2 (SENP2), a protease responsible for tuning the SUMOylation of proteins, suppresses stemness properties and promotes sorafenib sensitivity in HCC tumor model. In particular, SENP2 expression is reduced in HCC cells and in the CSCs with respect to its normal counterpart. Overexpression of SENP2 reduced the expression of stemness markers (i.e., CD133) and increased the sensitivity to sorafenib through the inhibition of AKT/GSK3β/CTNNB1 (β-catenin) signaling pathway. In cancer cell plasticity the tumor microenvironment (TME) plays a crucial role by different mechanisms, such as exosome-based intercellular communication. Lin et al. demonstrated the effects of exosomal miR-4800-3p in affecting epithelial-mesenchymal transition (EMT) and stemness properties of HCC cells by the regulation of the Hippo signaling pathway. Likewise, Shang et al. reported that exosomes derived from mesenchymal stem cells transmit TMBIM6 (Bax inhibitor-1) to promote EMT and stemness through activation of PI3K/AKT pathway in HCC cells. Among potential therapeutic approaches, Yao et al. reported that overexpression of miR-186 impairs the self-renewal capacity of CSCs in liver cancer by targeting PTPN11 (also known as SHP2), a protein tyrosine phosphatase of RAS/MAPK signaling pathway. Interestingly, miR-186 overexpression sensitizes HCC cells to cisplatin treatment.

On the other hand, targeting the molecular players of CSC trans-differentiation capacity could represent a promising anticancer strategy, particularly in tumors such as glioblastoma (GBM). Indeed, the GBM–endothelial cell trans-differentiation process represents one of the most recent GBM-associated neo-vascularization mechanisms that has been described, consisting of the direct trans-differentiation of GBM stem cells (GSCs) into endothelial cells. Targeting GSC trans-differentiation could represent a chance to overcome therapeutic resistance to anti-angiogenic treatment (5). Han et al. reported that the transformation process of GSCs to endothelial cells is regulated by the signal axis P4HA1/COL6A1 as an adaptive response to hypoxia, offering a novel target for anti-vascular therapy in GBM patients.

Triple-negative breast cancer (TNBC) is the most lethal subtype of breast cancer characterized by hyperproliferative cells lacking expression of estrogen receptor (ER), progesterone receptor (PR), and expression/amplification of human epidermal growth factor receptor 2 (HER2). It has been suggested that the presence of CSCs in TNBC significantly contributes to highly invasive and therapy resistance features, and recurrence. In particular, CD44, CD24, and aldehyde dehydrogenase 1 (ALDH1) are widely used markers for identifying CSCs in breast cancer (6). In their article, Chen et al. provided evidence that nucleolar and coiled-body phosphoprotein 1 (NOLC1), a nucleolar protein acting as a chaperone that shuttles between the nucleus and the cytoplasm and plays an important role in rRNA synthesis and in the regulation of tumorigenesis, is significantly associated to stemness properties of TNBC. The knockdown of NOLC1 inhibited mammosphere-forming capacity and decreased the expression of several stemness regulators (such as ALDH and MYC). Since the expression of NOLC1 is significantly different between TNBC and non-TNBC patients, this protein may represent a suitable target to reverse TNBC phenotype.

Myxoid liposarcoma (ML) is a subtype of liposarcomas, rare mesenchymal neoplasms arising from adipocytes. ML is characterized by the presence of FUS-DDIT3 gene fusion (in >95% of cases) as a result of the chromosomal translocation t(12;16)(q13;p11) (7). Dolatabadi et al. provide evidence that FUS-DDIT3 fusion oncoprotein increased the activation of the JAK-STAT signaling pathway that regulates stemness properties (down regulation of CD44) and chemotherapy resistance in ML.

Organoids are three-dimensional cellular complexes grown *in vitro* which maintain phenotypic, genetic, structural, and functional features of the original tissues. The use of organoids to study CSC differentiation has been proposed in recent years (8). Wang et al. reviewed the current and potential applications of organoids in tumorigenesis and tumor vaccination. The authors also focused on the possibility to use organoids for studying bidirectional communication between tumor cells and TME, for example, the crosstalk between cancer-associated fibroblasts and CSCs.

## Author contributions

Writing, Review, and Editing: MB, SB, and CT. All authors contributed to the article and approved the submitted version.
